# Risk Factors Associated With Hypertension in Young Adults: A Systematic Review

**DOI:** 10.7759/cureus.37467

**Published:** 2023-04-12

**Authors:** Meghanad Meher, Sourabh Pradhan, Soumya Ranjan Pradhan

**Affiliations:** 1 General Medicine, Institute of Medical Sciences (IMS) and Sum Hospital, Siksha 'O' Anusandhan (SOA) Deemed to be University, Bhubaneswar, IND

**Keywords:** young adults, smoking, risk factors, prevention, hypertension

## Abstract

On a global scale, hypertension ranks third among the six major risk factors for cardiovascular disease. The risk of heart disease, stroke, and renal failure is all significantly increased by hypertension. We looked for papers on risk factors associated with hypertension in young adults on Google Scholar and PubMed. “Hypertension,” “young adults,” and “risk factors” were the search terms. Eligibility testing was done in a standardized, non-blinded way. The first author, year of publication, subject related to hypertension in young adults, and risk factors associated with hypertension in young adults were all retrieved from each paper. A PubMed search yielded 150 results. In all, 10 papers were considered in our review, which were published between 2017 and 2021. Most of the studies considered were carried out by foreign research groups. Adults who smoke, chew tobacco, drink alcohol, are obese, engage in sedentary behavior, consume too much salt, and have unhealthy lifestyles are at a higher risk of developing hypertension. In addition to these risk factors, there were additional important risk variables such as illiteracy, illness ignorance, a disregard for one’s health, and a society that values men more than women. The way of life is radically altering because of people adjusting to Western culture. Smoking, drinking, being overweight, and eating too much salt are the primary risk factors for hypertension. This shows that in order to live a happier and healthier life, it is important to increase people’s understanding of and attitudes toward the prevention and control of hypertension.

## Introduction and background

In the past several decades, hypertension in young adults (18-24 years) and middle-aged people (25-44 years) has emerged as a serious issue for public health across the world. One of the leading causes of death and disability worldwide is the rising prevalence of hypertension [1]. A total of 9.4 million fatalities per year are attributed to hypertension worldwide [2]. In India, 28% of adults suffered from uncontrolled hypertension in 2008 [3]. More than 1.56 billion individuals will have hypertension by 2025, according to estimates, increasing the worldwide burden of this disease over the next decades [4]. One of the leading causes of death in the Western world, hypertension may also trigger strokes, renal problems, and even kidney failure. High blood pressure was a factor in the deaths of almost 7.6 million persons worldwide in 2001. This represents around 13.5% of all fatalities [3,5].

Due to its lack of severe symptoms and potentially fatal effects, hypertension has been known as a silent killer. The necessity of hypertension screening and treatment at an early stage cannot be overstated [6]. According to a recent national census, 15.2% of adults in Saudi Arabia have hypertension, and 57.8% of those cases are undetected. In a wealthy nation where healthcare is readily available at no cost, this comes as a shock [7]. Early detection and adequate therapy of these instances may avoid future consequences; this is true, even though the incidence of hypertension is lowest in young individuals compared to middle-aged adults and the elderly [8,9]. Hypertension is quite common among Chinese individuals aged 18 and above [10]. Although it is more prevalent in the elderly, a growing prevalence in the youth population is being recorded [11]. However, the incidence of hypertension diagnoses among young individuals who fit the criteria for the diagnosis is lower than among middle-aged and older persons [12]. For this reason, it may be useful to focus public health preventative efforts on young people who exhibit a higher risk of developing hypertension. Also, hypertension management is easier and takes less time for young persons to attain than it does for older ones [13].

Additionally, prehypertension, which is more common in young people than full-blown hypertension, is a major antecedent for developing hypertension and cardiovascular disease later in life, and when diagnosed early, it may be decreased, although not always effectively, by lifestyle adjustments [14,15]. Despite this, there is a deficiency in prehypertension and hypertension screening among young people, and doctors are less likely to prescribe antihypertensive drugs to young adults with hypertension than they are to older patients. Also, because hypertension may be prevented and improved blood pressure management can be attained by the reduction of risk factors, this is an essential tool. Diets heavy in salt, excess body fat, lack of exercise, inadequate consumption of fruits and vegetables, and excessive alcohol use all increase the risk [16]. The incidence of hypertension among future doctors has been the subject of much research. Globally, hypertension has been the subject of several epidemiological research [17]. Stroke, coronary heart disease, congestive heart failure, and chronic kidney disease or decreased renal function are only a few of the numerous severe diseases linked to elevated blood pressure, according to these research [18]. Young people are particularly vulnerable to hypertension because of risk factors including smoking and frequent drunkenness [19]. Few studies have shown no link between hypertension and working out [20]. However, hypertension is linked to the kinds of inactivity that were mentioned [21].

## Review

Materials and methods

Source of Data and Eligibility

We devised a search method to find articles that would be included in the systematic review. Searches for possibly suitable papers were conducted using electronic databases such as PubMed. In addition, online databases including Google and Google Scholar were employed to find any possible eligible papers.

The review included (a) studies focused on hypertension in young adults and (b) peer-reviewed journal articles published between January 2017 and March 2020 that were written in English. Following that, articles were examined and rated independently, and data was extracted from them. Other exclusion criteria included publications that did not include original research (such as abstracts, reviews and perspectives, comments, and letters to the editor) and papers written in languages other than the English language.

Screening Strategy

Following a review of the titles and abstracts of obtained studies from the relevant electronic databases, the words used in the initial search were categorized into four categories and merged with the Boolean operators “AND” and “OR,” while the search process used electronic databases mentioned above. The entire texts of each paper were downloaded and examined, and only the papers that passed the screening process were included in the study. When complete texts of relevant papers were not available or unavailable, the authors of the papers were requested to provide them. If it was not feasible to contact the writers, for example, because of a nonresponse or a negative response, the entire manuscript was bought. In addition, the reference lists of the relevant papers were examined to increase the chances of finding publications that were suitable for inclusion. To represent the complete sequential process of the screening approach, the Preferred Reporting Items for Systematic Reviews and Meta-Analyses (PRISMA) flowchart was utilized.

Data Verification for Consistency

An Excel spreadsheet (Microsoft Corp., Redmond, WA, USA) containing the relevant data was created to ensure the database’s internal quality control. The information was also reviewed for integrity as part of the database’s external quality control process. When mismatches in Excel sheets occur, the data were examined again.

Result

**Figure 1:**
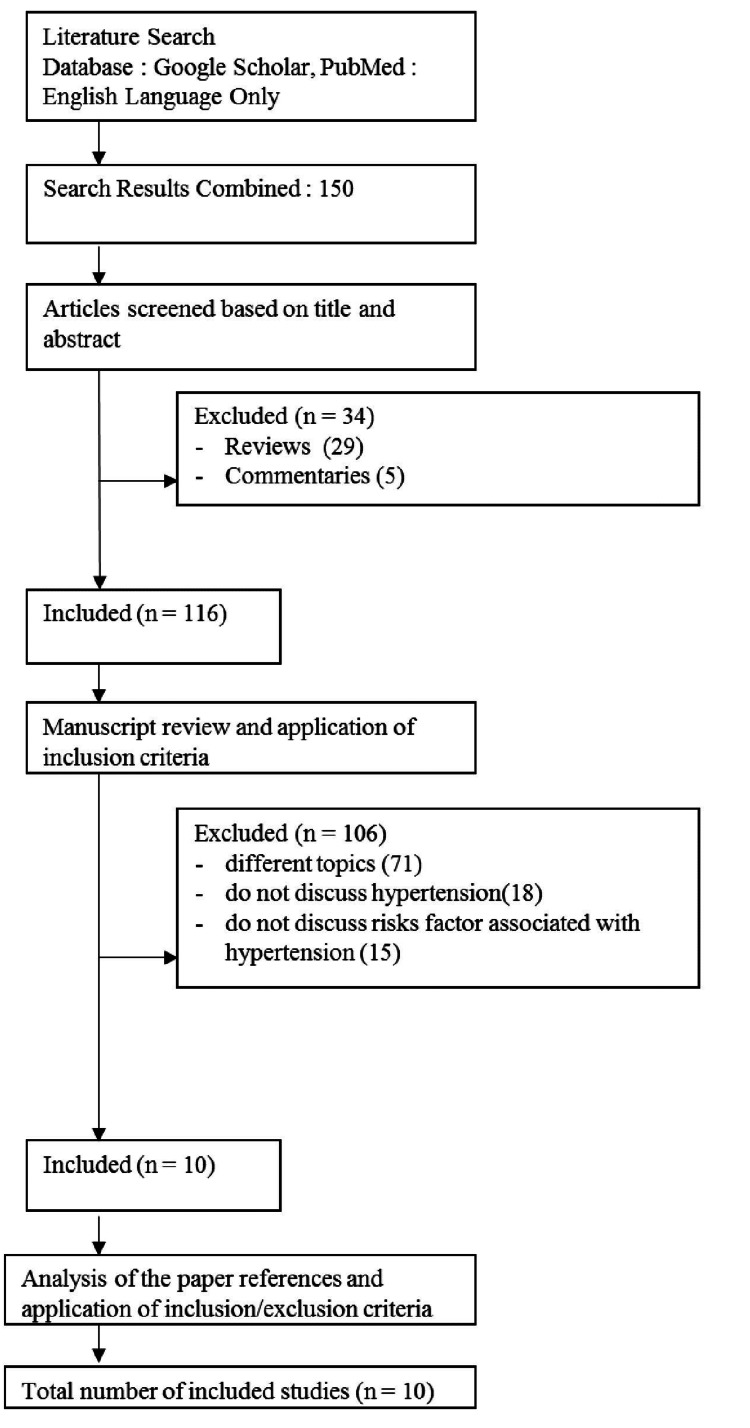
PRISMA flowchart PRISMA: Preferred Reporting Items for Systematic Reviews and Meta-Analyses

Figure 1 shows the PRISMA flowchart indicating the specifics of the search.

Result of the Search

We looked at 200 sources, 196 of which were through database searches and 14 from other sources. The total number of references for screening was 144 after duplicates were removed. The first step of screening produced papers based on title and abstract. All 144 studies were subjected to another level of full-text assessment. We eliminated studies that did not meet the study’s qualifying requirements. As a result, the review comprised 10 studies. Table 1 provides an overview of the papers included in the review.

**Table 1:** Studies included in the review BMI: body mass index, CI: confidence interval, OR: odds ratio, BSU: Bishop Stuart University

Author	Sample size	Area	Main findings
Singh et al., 2017 [22]	640	Varanasi	About a third of the participants had high blood pressure, while half were at risk for developing hypertension. Low levels of education, care, and regulation of hypertension were also prevalent.
AlWabel et al., 2018 [23]	130	Saudi Arabia	Gender, BMI, and previous diagnoses of diabetes and hypertension were shown to be significantly linked. Their findings provide credence to the widely held belief that young individuals have a disproportionately high prevalence of prehypertension and hypertension, the vast majority of whom remain untreated.
Hu et al., 2017 [24]	15,296	China	There is a widespread pandemic of prehypertension and hypertension in southern China. More research is required to identify an indicator that correctly represents visceral fat and is closely related to cardiovascular disease.
Ondimu et al., 2019 [25]	160	Kenya	Individuals with a BMI of 25 or below were 3.05 times more likely to be hypertensive (95% CI: 1.26, 7.40; p=0.014). The risks of developing hypertension almost tripled if you had a blood pressure-raising relative (OR: 2.78, 95% CI: 1.20, 6.46; p=0.018). The risk of developing hypertension was cut by almost 70% in those who gave up alcohol.
Liu et al., 2017 [26]	4,120	China	Findings showed that hypertension rates among local youth increased between 2011 and 2014. Hypertension is most common in men aged 40 and over, those with lower socioeconomic status who work in rural regions, those who are overweight or obese, and those who smoke cigarettes.
Paul et al., 2020 [27]	322	Bangladesh	The main modifiable risk factors of hypertension in young people (18-44 years)include tobacco use, obesity, dyslipidemia, and excessive salt consumption. These variables, together with antihypertensive drugs, are essential for the prevention and treatment of hypertension.
Amanyire et al., 2019 [28]	156	Uganda	The BSU faculty and staff are not immune to the dangers of hypertension, diabetes, and obesity, but there is a lack of information about their causes, symptoms, and how to avoid them. Public health measures for the university workers include regular exercise, regular checkups, and a regulated diet.
Tymejczyk et al., 2019 [29]	61,000	Haiti	The rates of hypertension, obesity, and unmet healthcare requirements were all quite high across all age groups. The advantages of more cost-effective preventive and treatment programs should be extended to slum inhabitants; therefore, a deeper understanding of the links between intraurban migration and environmental risk factors for hypertension is necessary.
Mouhtadi et al., 2018 [30]	1,362	Lebanon	Although hypertension is common among adults in Lebanon due to a number of factors, these causes are often not fully appreciated, resulting in inadequate management of the condition. Young adult men (aged 18-29) had the highest prevalence of hypertension. These results highlight the urgent need for better hypertension identification, treatment, and management among adult populations in Lebanon.
Bui Van et al., 2020 [31]	319	Vietnam	High hypertension prevalence was found in two Chiem Hoa District communes in Tuyen Quang province. Overall hypertension and isolated systolic hypertension risk variables were age, BMI, waist-to-hip ratio, and inactivity. The effects of ethnicity on isolated systolic hypertension were particularly notable.

Discussion

There are a few dissimilar findings, but overall, the results from the chosen research are rather similar. Smoking, drinking, being overweight, having a family history of hypertension, getting older, eating a diet high in salt, developing diabetes, and having a body mass index (BMI) above 30 kg/m^2^ are some of the major risk factors identified for the development of hypertension in young and middle-aged Indian adults. Waist-to-hip ratio, socioeconomic status, central obesity, high cholesterol, inactivity, poor nutrition, lack of exercise, lack of education, lack of knowledge, and a history of vascular disease are all factors that have been found in the research. The following sections provide context for these results by discussing and comparing them to the relevant literature and other accessible information.

Excessive Salt Intake

Consuming an unhealthy amount of salt is a leading cause of high blood pressure in both adults and older people. Several of the analyzed studies revealed this risk factor to be present. Those who eat more than 10 g of salt per day in their diet are at an increased risk of developing hypertension, according to research conducted in a variety of geographical areas [27]. An excessive intake of salt was identified as a major risk factor in these investigations (p<0.001) due to its prevalence among the study participants. The average Asian diet has higher salt than the average Western diet, according to a study by Singh et al. in 2017 [22]. The Indian community as a whole would benefit from measures and awareness aimed at lowering their salt intake since salt consumption has rapidly become a key risk factor in the country.

Alcohol Consumption, Tobacco, and Smoking

Among adults, the most notable modern-day risk factors are alcohol, cigarettes, and smoking. These risk factors have been revealed to be more responsible for the development of hypertension in studies conducted by Singh et al. (2017) [22], Tymejczyk et al. (2019) [29], and Paul et al. (2020) [27]. The two major risk factors among young adults are alcohol use and tobacco use. In the research conducted by Singh et al. (2017) [22], the p value for cigarette smoking was 0.01 and that for alcohol intake was 0.001. Tymejczyk et al. (2019) [29] found that among the participants, 88% smoked cigarettes and 54% drank alcohol. People in Bangladesh use tobacco for both smoking (46%) and chewing (13.7%), as reported by Paul et al. (2020) [27]. Tobacco chewing was quite prevalent among both sexes of the subjects. The study results revealed that both young and adult smokers were at increased risk for hypertension while using any kind of tobacco.

Obesity/BMI/Waist-to-Hip Ratio

Obesity, overweight, high body mass index (BMI), and a high waist-to-hip ratio are risk factors for hypertension and other noncommunicable illnesses. The waist-to-hip ratio and body mass index (BMI) are the standard methods for determining a person’s degree of overweight or obesity. For young women, having a high body mass index (BMI > 25 kg/m^2^) is a major risk factor for developing hypertension, as shown in the studies by Singh et al. (2017) [22], AlWabel et al. (2018) [23], Hu et al. (2017) [24], Ondimu et al. (2019) [25], Liu et al. (2017) [26], Paul et al. (2020) [27], Amanyire et al. (2019) [28], and Tymejczyk et al. (2019) [29]. Most of the patients in their research had a high body mass index (BMI > 25 kg/m^2^) or a large waist-to-hip ratio (>0.85), and 58% of the participants in the study had hypertension [23-28].

Diet and Physical Fitness

One cause of high blood pressure is the country’s unhealthy diet and growing love of fast food. In addition, the rising consumption of oil and fatty foods in recent years may be contributing to an increase in the prevalence of obesity, as shown by the research of Bui Van et al. (2020) [31]. The majority of patients with hypertension have a poor diet that is high in saturated fat and low in protein and carbohydrates [31]. Having fewer fruit and vegetables in one’s diet has been linked to an increase in hypertension, as shown by the research of Liu et al. (2017) [26]. The way of life and a lack of exercise are also major contributors to the development of hypertension [26]. The majority of participants in these research had sedentary lifestyles, consuming large amounts of alcohol, smoking often throughout the day, and eating fewer fruits and vegetables. Since being overweight increases one’s risk of getting hypertension, eating a diet high in oily and fatty foods, engaging in less physical activities, and not exercising may have a significant impact. Awareness programs regarding good eating are essential to prevent this.

Family History

It is a usual practice for parents to pass on their traits to their kids via generations. There are a lot of illnesses that may be passed on from parents to kids. Their kids may or may not be affected by these disorders. The exact mechanism by which this exchange takes place is currently being studied by scientists. Diseases may manifest in the progeny at any time, some in infancy and others in old age. According to AlWabel et al. (2018) [23], hypertension is also a heritable condition. Children of parents with hypertension are more likely to get the condition themselves. As a result, a kid is at a higher risk of developing hypertension if both of his or her parents have the condition and if the child is exposed to additional risk factors, such as smoking, drinking alcohol, eating a high-fat diet, and leading a sedentary lifestyle [24]. According to the research by Ondimu et al. (2019) [25], there is a significant link between hypertension and genetics (p<0.001). About a quarter of hypertensive individuals in the research had a family history of the disease, and they were also less likely to be exposed to the other risk factors. All participants in this research were adult males between the ages of 20 and 49, and 58% of them had hypertension [27,30]. Together, obesity and family history constituted their greatest risk. This fits well with the links between fat and hypertension seen in families. There were many overweight and obese young women in this research. Additionally, they have a predisposition for hypertension due to a history of the disease in their family. Hypertension in the family is somewhat understood, yet further study and specifics are required for a full understanding. More research and understanding will allow us to cut off the transmission of these illnesses at their genetic source, saving many lives and preventing countless disabilities.

Sex

Exactly how sex affects blood pressure is not simple to correctly identify. We all know that there are significant physiological, psychological, and hormonal variations in the ways in which men’s and women’s bodies work. The analyzed papers by Mouhtadi et al. (2018) [30] indicated that hypertension mostly affects women. Hypertensive patients accounted for 399 of the total population. Since most rural women are stay-at-home mothers, it is possible that there are more female hypertension patients within the Lebanese young adult population where this research was conducted. Their risk of getting high blood pressure increases since they are sedentary and seldom leave the house. These elements may have a greater impact on the rising prevalence of hypertension and other noncommunicable diseases (NCDs) in women [30]. Both AlWabel et al. (2018) [23] and Liu et al. (2017) [26], however, reported a higher male-to-female ratio. Participants may be to blame for the disproportionate number of men in these research. Substance abuse and tobacco use were the most modifiable risk variables among the study participants. Tobacco use and alcohol use are more common among men in India. In male patients, these variables may have contributed to the onset of hypertension [23,26].

Illiteracy

The research by Singh et al. (2017) [22] was conducted in the Varanasi area of India. More people in the urban population were illiterate, and this was the most significant risk factor for hypertension. In India, hypertension and other noncommunicable illnesses face a significant barrier in the form of widespread illiteracy. In most nations, it contributes to illiteracy, undernourishment, and both short- and long-term health problems. Poverty and a lack of education are widespread issues in India and other developing nations. Each of these variables influences the likelihood of the others. The government’s established educational programs are not being properly managed or standardized. Furthermore, as was previously established, male dominance culture is to blame for the higher illiteracy rate among women and their increased susceptibility to sickness. The Indian populace may benefit from increased educational opportunities if these illnesses are to be controlled. The prevalence of illness and the rate of premature mortality may be reduced by addressing poverty and improving health literacy.

Socioeconomic Status

In addition to acting as a risk factor for hypertension, a person’s socioeconomic level may also be a cause of it. According to Singh et al. (2017) [22], the majority of Indians with hypertension are from the middle class and upper class. Obesity, hypertension, and other noncommunicable illnesses are more prevalent in these populations. Men and women are equally represented and at risk in this hypertension class. Because many middle- and upper-class jobs require little physical exertion, they may be at greater risk than lower-income workers. It is also possible that those in higher socioeconomic groups have better health because of their access to better food and more leisure time. However, this may cause them to gain weight, which increases their risk of developing hypertension and other health problems including heart disease and stroke. Nevertheless, hypertension is not exclusive to those with more means. Most persons in lower socioeconomic brackets lack access to nutritious diets and have inadequate knowledge about hypertension. Hypertension may be caused by a lack of potassium in the diet and an increase in salt consumption, both of which can be avoided by increasing the regular intake of fruits and vegetables. The lack of education and knowledge about the dangers of the illness further increases their vulnerability.

Strength and limitations

Although there was a limitation of prior research on this issue, the search procedure outlined in the methodology section was thorough. The procedure was adhered to in terms of the number of times articles and evidence were assessed and the inclusion/exclusion criteria used. Although great care was taken to minimize the possibility of bias and maximize the reliability of the results, there were still some shortcomings in the research. Young individuals (ages 18-50) comprised the study sample; nevertheless, research targeting this age range has been scarce, particularly in the realm of hypertension and associated risk factors. Since India is both a large nation and a diverse society, it is possible that the findings of these research may not apply to the population as a whole. Some of the examined studies might have been flawed due to unclear inclusion/exclusion criteria, muddled analyses, and vague conclusions. The sample sizes of certain research were also insufficient to draw broad population-level conclusions.

## Conclusions

Hypertension is an overlooked problem in young adults. Young individuals have an underrecognized hypertension issue. The research indicated that hypertensive young people had higher rates of cigarette use, obesity, dyslipidemia, and excessive salt consumption than the general population. In addition to taking medication, addressing these variables is essential for preventing and effectively managing hypertension. The findings of this study are encouraging since they point the way toward improved methods of treating hypertension and its risk factors. Full implementation of the National Programme for Prevention and Control of Cancer, Diabetes, Cardiovascular Diseases, and Stroke (NPCDCS) and other national or worldwide level programs is necessary to address all aspects of hypertension and its problems, with an emphasis on young people.
